# Transsynaptic modality codes in the brain: possible involvement of synchronized spike timing, microRNAs, exosomes and epigenetic processes

**DOI:** 10.3389/fnint.2012.00126

**Published:** 2013-01-04

**Authors:** John Smythies, Lawrence Edelstein

**Affiliations:** ^1^Center for Brain and Cognition, University of California San DiegoLa Jolla, CA, USA; ^2^Medimark CorporationDel Mar, CA, USA

**Keywords:** molecular codes, synchronized oscillations, signaling molecules, microRNAs, exosomes

## Abstract

This paper surveys two different mechanisms by which a presynaptic cell can modulate the structure and function of the postsynaptic cell. We first present the evidence that this occurs, and then discuss two mechanisms that could bring this about. The first hypothesis relates to the long lasting effects that the spike patterns of presynaptic axons may exert by modulating activity–inducible programs in postsynaptic cells. The second hypothesis is based on recently obtained evidence that, the afferent neuron at the neuromuscular junction buds off exosomes at its synapse and carries a cargo of Wg and Evi, which are large molecular transsynaptic signaling agents (LMTSAs). Further evidence indicates that many types of neurons bud off exosomes containing payloads of various lipids, proteins, and types of RNA. The evidence suggests that they are transmitted across the synapse and are taken up by the postsynaptic structure either by perisynaptic or exosynaptic mechanisms, thus mediating the transfer of information between neurons. To date, the molecular hypothesis has been limited to local interactions within the synapse of concern. In this paper, we explore the possibility that this represents a mechanism for information transfer involving the postsynaptic neuron as a whole. This entails a review of the known functions of these molecules in neuronal physiology, together with an estimate of the possible types of information they could carry and how they might affect neurocomputations.

## Introduction

In this paper we address the general question of how presynaptic cells can exert long-lasting influences on their projection targets and thus affect the modality identity and properties of specific cortical regions. To answer this, we propose two general solutions. The first is that, the spike patterns of the synaptic axons may exert long-lasting effects by modulating activity–inducible gene expression programs in postsynaptic cells, with different spiking patterns possibly eliciting the expression of different cohorts of epigenetic signaling molecules. The second is that, presynaptic cells exert effects through the transsynaptic transmission of a variety of signaling molecules, possibly through exosome-related mechanisms. We will start by considering the evidence that presynaptic cells do influence postsynaptic cells in this manner.

## The role of the presynaptic neuron in determining modality: the effect of deafferentation and reafferentation

We can approach this topic by asking, what determines the modality of a sensory neuron e.g., whether activation of such a neuron leads to a visual or to a somatosensory experience. During embryogenesis, the newly differentiated cortical areas each secrete specific attractant molecules that guide the incoming thalamic relay axons to the correct location. In normal circumstances during later development into adulthood these connections maintain their overall pattern although subject to a deal of synaptic plasticity (Smythies, 2002). The degree to which this is maintained during later stages of growth by signaling and epigenetic factors transmitted from the presynaptic to the postsynaptic neuron is currently unknown. However, under extreme conditions of massive deafferentation and reafferentation, this system can undergo more extensive changes. For example, in experiments on blind subjects skilled in Braille. Ptito et al. (2008) showed that magnetic transcranial stimulation of neurons of loci in the optic cortex results in a somatosensory, and not a visual, experience in these subjects (Ptito et al., 2008). In these cases, some differentiated “visual” cells are “taken over” by the somatosensory system, and start to process somatic information instead. This activity generates somatosensory sensations in consciousness (sensations that the fingers are being touched) in place of the normal visual sensations. These authors conclude: “Our data show that the qualitative character of the subject's experience is not determined by the area of cortex that is active (cortical dominance), but by the source of input to it (cortical deference).”

At a functional level, a deafferented cortex (e.g., the visual cortex in the blind) can take over functions of another sensory modality (e.g., hearing), but only if the functions of the two are homologous. That is, spatial hearing functions are improved, but not tone discrimination (Lomber et al., 2010). If the auditory cortex is deafferented as in the deaf, then visual spatial discrimination is improved, but not color functions. By employing an experimental design that allows independent control of both spatial and contextual correspondence, Heron et al. (2012) showed that observers are able simultaneously to adapt to two opposing temporal relationships, provided they are segregated in space. No such recalibration was observed when spatial segregation was replaced by contextual stimulus features (in this case, pitch, and spatial frequency). The authors suggest that these effects provide support for dedicated asynchrony mechanisms that interact with spatially selective mechanisms early in visual and auditory sensory pathways. However, this account of “partial” restructuring leaves unanswered the question of whether the reafferented cortex should be regarded as having a new modality, or having undergone a modulation of its modality. We prefer to leave this matter open at this stage. In either event, the data indicates that the presynaptic neuron exerts a powerful influence over the structure and function of the postsynaptic neuron that must be transmitted by some form of code or agent.

Another source of information about sensory modality determination comes from sensory input rerouting [diverting the sensory inflow of one system to the cortex of another system by neonatal diversion of e.g., retinal axons to the auditory thalamus (cross-modal rewiring)]. This operation leads to profound changes on diverse components of cortical circuitry, both at the anatomical and functional levels (Gao and Pallas, 1999; Sharma et al., 2000). Sensory input rerouting can also lead to changes in intracolumnar information processing in the postsynaptic neuron. For example, if the input to an auditory neuron in the cortex is replaced by a visual input, then the response characteristics of information processing in its cortical columns changes so as to mimic the systems employed in a visual neuron. These auditory cortical cells develop visual response properties such as direction selectivity, orientation tuning, and simple/complex receptive-field structure (Roe et al., 1992). The auditory cortex also develops retinotopic maps [Roe et al., 1990; and see Linden and Schreiner (2003) for a comprehensive review]. Rerouting visual inputs to the auditory thalamus can also reorganize callosal connections in the auditory cortex, causing both a reduction in their extent and a reorganization of the pattern (Pallas et al., 1999). Chowdhury and De Angelis (2008) have extended the range of cortical plasticity by showing, in depths perception discrimination experiments, that the contribution of particular brain areas to task performance can change dramatically as a result of learning new tasks.

These changes would appear to be the result of some signaling system in the afferent axons. This implies that these axons are carrying a code that is most probably contained either in the spatiotemporal patterns of its spike trains, or by some form of molecular or epigenetic signal, or both. The details of how these modality-related codes systems have such remarkable effects are not currently known. The aim of this present paper is to enquire what form this code might take and how it could bring about the extensive effects on the postsynaptic neuron reported. We should make it clear by “epigenetic factor” here we mean first order molecules, such as specific proteins and RNAs. We do not include, for our present purposes, transcription factors nor processes like methylation and acetylation. In this paper, moreover, we have focused, not on modality code adaptions to extreme conditions, but to their nature under ordinary brain activities.

## Differentiation of neuronal changes following acute and chronic deafferentation

Acute deafferentation induces functional changes in the brain's networks, whereas chronic deafferentation also results in structural changes. Rerouting sensory pathways in the brain involves sensory deafferentation since one part of the cortex is deprived of its normal input. Certain cases of sensory deafferentation also involve transmodal rerouting of pathways, as when the deafferented neurons get invaded by axons belonging to another modality. This has both immediate and delayed effects on the sensory system in the brain concerned. The immediate effects are due to dynamic changes in brain networks.

Delayed effects, however, involve much rewiring. Recent studies show that, in the long term, substantial reorganization in subcortical structures, including the brainstem and thalamus, occurs that may be of sufficient extent to account for, or play a large part in, representational plasticity in somatosensory cortex (Jones, 2000). Extensive interhemispheric corticocortical reorganization can occur in the rodent brain following peripheral nerve deafferentation (Pelled et al., 2007). FMRI data shows that long-term reorganization of the somatosensory cortex, following spinal cord injury in humans, is associated with changes in local cortical anatomy and provide “compelling evidence” that such reorganization in humans results from the growth of new lateral connections from adjacent cortex into the deafferentated portion, and not simply from the unmasking of already existing lateral connections (Henderson et al., 2011)

Olfactory deafferentation induces whisker tactile hypersensitivity. In studies in mice 1 week after olfactory deafferentation, Ni et al. (2010) showed that, there results a recruitment of more GABAergic neurons and their fine processes in the barrel cortex, as well as an up-regulation of their capacity to encode action potentials. The hyperpolarization driven by inhibitory inputs strengthens the encoding ability of their target cells.

Churchill et al. (2004) conducted Golgi studies in the somatosensory cortex in primates following deafferentation. They showed that, after denervation, there is a systematic change in the dendritic arborization pattern of both layer II/III pyramidal and layer IV spiny stellate cells in the contralateral hand region of area 3b, compared to unaffected cortical areas. This was marked by a progressive expansion of distal regions of the dendritic arbor, both basilar and apical, with no appreciable changes proximally.

Recently considerable attention has been paid to the role of single neurons, particularly their dendritic arbors, in information processing (London and Häusser, 2005; Gollo et al., 2009; Klausberger, 2009; Branco et al., 2010). In particular a paper by Legenstein and Maass (2011) is relevant to the subject of this review. They propose that non-linear processing in dendritic branches endows individual neurons with the ability to perform complex computational operations necessary to solve for example the binding problem. They investigated how experimentally observed plasticity mechanisms in dendritic arbors, such as depolarization-dependent spike-timing-dependent plasticity and branch-strength potentiation, could be integrated to conduct self-organized nonlinear neural computations with dendritic spikes. It seems possible, therefore, that changes in dendritic arbor neurocomputations may be involved. Changes in the spatial arrangement of interlaminar glia may be an integral part of the long-term process of structural reorganization of the cerebral cortex following cortical deafferentation (Reisin and Colombo, 2004).

## Spike train modality codes

Spikes, and bursts of different durations, code for different stimulus features. The biophysical mechanism of spike generation enables individual neurons to encode different stimulus features into distinct spike patterns (Kepecs and Lisman, 2003). Cortical regular-spiking neurons can propagate filtered temporal information in a reliable way through the network, and with high temporal accuracy (Asai et al., 2008). Since the changes induced by deafferentation can transfer across synapses (as when a new input to the thalamus induces such changes in the cortex) the mechanism that does this must involve transsynaptic transfer. The brain is a highly modular structure. Therefore, spiking activity must be able to propagate from one module to another while preserving the information it carries (Kumar et al., 2010). If the brain uses spike timing as a means of information processing, other neurons receiving spatiotemporal spikes from such sensory neurons must also be able to treat information included in the inter-spike intervals (Masuda and Aihara, 2002). Significant amounts of visual information are represented with high precision by details of the spike train at millisecond and sub-millisecond precision (Nemenman et al., 2008). Diesmann et al. (1999) state that precisely synchronized action potentials can propagate within a model of cortical network activity that recapitulates many of the features of biological systems. Axons can carry multiple codes in the spatiotemporal patterns of their spike trains (Kayser et al., 2009). Singer (2009) proposes that axons can carry two messages in parallel. The first indicates the presence of the feature to which their neurons are tuned. The second conveys the information with which other neurons (specific target cells or members of a coherent assembly) they are communicating. The first message is carried by a rate code. Singer proposes that the second code is a function of the precise timing relationships between individual spikes of distributed neurons (temporal code). These relations are established, he suggests, either by the timing of external events (stimulus locking), or by internal timing mechanisms based on an oscillatory modulation of neuronal responses in different frequency bands. Therefore, we need to discover what the individual spike codes are that carry the modality information. We could find only one paper in the literature on this topic. A direct investigation of modal codes in the cortex has shown that neuronal spiking patterns are regular in motor areas, random in the visual areas, and “bursty” in the prefrontal area (Shinomoto et al., 2009). Supporting evidence in part for our hypothesis has been obtained by Yang and Zador (2012), who carried out experiments on rats measuring their ability to discriminate minimum timing differences of electrical stimuli delivered to different cortical modalities. They found wide differences ranging from 1 ms in barrel cortex to 15 ms in visual cortex. They concluded that, different cortical areas are adapted to the specific structure of the input signals they process, and that precise spike timing may play a more important role for some cortical areas than for others.

There is also a need to find out how these spike codes produce their transsynaptic effects. We could find no papers published that tackle this subject.

## Evidence for the activity of large molecular transsynaptic signaling agents (LMTSAs) in neural communication

Many types of cells, including neurons, bud off exosomes from their plasma membranes into the extracellular environment. Exosomes are small lipoprotein vesicles derived from the intraluminal membranes of multivesicular bodies (MVB) of the endocytotic pathway. They are expelled into the extracellular space upon fusion of the MVB with the plasma membrane (Fauré et al., 2006). Endocytosis is a similar process, acting in the other direction. This transports many activated membrane receptors into the cell (Smythies, 2002). However, for long the only form of exocytosis recognized in neurons was the familiar synaptic vesicle neurotransmitter and neuromodulator system. Exosomes carry molecules between cells. In a recent review, Tetta et al. (2012) state “Extracellular vesicles, including exosomes and microvesicles, may deliver lipids and various functional transcripts, released from the cell of origin, to target cells. Since extracellular vesicles contain defined patterns of mRNA, microRNA, long non-coding RNA, and occasionally genomic DNA, they may transfer genetic information which induces transient or persistent phenotypic changes in recipient cells”. In another recent paper, O'Loughlin et al. (2012) state “Exosomes play an important role in endogenous cell-to-cell communication … [and have been]… shown to be capable of traversing biological barriers and to naturally transport functional nucleic acids between cells.” Koles and Budnik (2012) conclude, “Exosomes, small secreted microvesicles, are implicated in intercellular communication in diverse cell types, transporting protein, lipid, and nucleic acid cargo that impact the physiology of recipient cells.” Although the transsynaptic transport of such molecules in the case of neurons has been experimentally limited so far as to signaling proteins (Wg and Evi), it seems highly unlikely that neurons would be exceptions to the general rule that exosomes transport a variety of nucleic acids as well. We will use the term large molecular transsynaptic signaling agents (LMTSAs) to define these cargoes carried by exosomes. Potential LMTSAs include lipids, trophins, and morphogenetic proteins, mRNA and microRNAs, but not perinuclear transcription factors. The first line of enquiry that we will follow is to ask—what function do these agents have in neurons?

## Proteins

Afferent axons could initiate transsynaptic modulation by secreting agents similar to neuroserpin and doublecortin. Neuroserpin is an axonally-secreted protein member of the serpin superfamily of serine protease inhibitors, and is widely expressed throughout the cerebral cortex, hippocampus and amygdala (Berger et al., 1999; Lee et al., 2012). These authors report that neuroserpin mRNA is increased in cultured hippocampal neurons upon depolarization by means of elevated extracellular KCl. They further outline a neurochemical link between an artificially induced depolarization and neural plasticity, which could be followed, in the case of a natural synapse, if the afferent axon secreted neuroserpin or a similar agent. Neuroserpin has also been found to modulate the growth and shape of axons and dendrites in the hippocampus (Borges et al., 2010). The doublecortin family of proteins modulate microtubular function in developing neurons (Dijkmans et al., 2010).

Additional proteins of interest (e.g., Li cell adhesion molecule, GPI-anchored prion protein, Glu 2/3 receptor subunit, brain-derived neurotrophic factor, neurotrophins) will be discussed below.

## The role of microRNAs and exosomes

We feel that promising candidates for the role of molecular carriers of information codes so far lies in the recently developed fields of microRNAs. There is now considerable data to indicate that microRNAs modulate a number of neuronal functions. To give some examples:

– Cohen et al. (2011) have identified a developmentally and activity-regulated microRNA (miR-485) that controls dendritic spine number and synapse formation in an activity-dependent homeostatic manner.
– Transcription of the microRNA miP335 is promoted by naturally evoked synaptic activity at the climbing fiber-Purkinje cell synapse in the mouse cerebellar flocculus.
  The target mRNAs for this microRNA have been identified as calbindin and 14-3-3-theta (Barmack et al., 2010).
– Impey et al. (2010) report that, neuronal activity regulates spine formation, in part, by increasing miR132 transcription, which in turn activates a Rac1-Pak actin remodeling pathway.
– MicroR-181a activity in primary neurons, induced by dopamine signaling, is a negative post-transcriptional regulator of GluA2 expression (Saba et al., 2012). Additionally these authors report that, miR-181a overexpression reduces GluA2 surface expression, spine formation, and miniature excitatory postsynaptic current (mEPSC) frequency in hippocampal neurons. Thus microR-181a could regulate synaptic function.
– Utilizing a mouse line with a conditional neuronal deletion of Dgcr8, a microRNA biogenesis protein predicted to process microRNAs exclusively, Hsu et al. (2012) produced evidence that some microRNAs govern essential aspects of inhibitory transmission and interneuron development in the mammalian nervous system.

Most studies of microRNAs in neurons have concentrated on the effects of these agents on process in the same neuron that produced them. However, it is possible that microRNAs, as well as other factors, may be exported from one neuron by synaptic transfer to other neurons. One mechanism may be by syncytia composed of gap junctions. Another mechanism has been suggested in trail breaking papers by Fauré et al. (2006) and Smalheiser (2007, 2009) relating to exosomes.

Fauré et al. (2006) found that, exosomes released by cortical neurons contain the L1 cell adhesion molecule, the GPI-anchored prion protein, and the GluR2/3 but not the NR1 subunits of glutamate receptors. They also found that exosomal release is regulated by K+-induced depolarization. They concluded that exosomes might have a regulatory function at synapses. In a later paper using mature cortical neurons in culture, Lachenal et al. (2011) observed exosomes being released from the somatodendritic compartment of neurons. They also found that this process was modulated by glutamatergic synaptic activity, and similarly concluded that exosomes might take part in normal synaptic activity. In the most recent paper from this group at INSERM, Chivet et al. (2012) concluded: “Exosomes could thus represent an ideal mechanism for interneuronal transfer of information.” This is a conclusion with which we agree. Recently, Turola et al. (2012) state: “Microvesicles (MVs) [a.k.a. exosomes] are released from almost all cell brain types into the microenvironment and are emerging as a novel way of cell-to-cell communication.”

The L1 cell adhesion molecule, found in synaptic exosomes by Fauré et al. (2006), has many functions that might link its appearance in the postsynaptic neuron with structural modality modulation. For example, it is involved in axon guidance; it alters the expression of transcription factors in murine neocortex (Kishimoto et al., 2012); it facilitates dendritic and axonal compartmentalization (Winther et al., 2012); it regulates the development of septal cholinergic neurons (Cui et al., 2011); perisomatic GABAergic innervation in prefrontal cortex is regulated by ankyrin interaction with the L1 cell adhesion molecule (Guan and Maness, 2010); it acts transcellularly to promote synaptic maturation on the neurons in culture (Triana-Baltzer et al., 2008); and a close relative, neural recognition molecule close homolog of L1 (CHL1), has been shown to regulate the orientation of apical dendrites in the mouse cortex (Ye et al., 2008).

Smalheiser (2007, 2009) suggested that, exosomes are involved in much transsynaptic activity. He based his hypothesis on the observation that exosomes contain a mixture of proteins and RNAs including mRNAs and microRNAs (Ratajczak et al., 2007; Valadi et al., 2007). Furthermore, exosomes express cell recognition molecules on their surface, which facilitates selective targeting and their uptake into recipient cells. This led him to suggest that exosomal secretion of proteins and RNAs may be a fundamental mode of communication within the nervous system, supplementing the known mechanisms of anterograde and retrograde signaling across synapses.

Smalheiser goes on to say: “In one specific scenario, exosomes are proposed to bud from the lipid raft region of the postsynaptic membrane adjacent to the postsynaptic density, in a manner that is stimulated by stimuli that elicit long-term potentiation. The exosomes would then transfer newly synthesized synaptic proteins (such as CAM kinase II alpha) and synaptic RNAs to the presynaptic terminal, where they would contribute to synaptic plasticity.”

Note that here he is suggesting that the flow on information is from the postsynaptic neuron back to the presynaptic neuron. This may well be the case for certain functions. However, in addition, we suggest that, to explain fully the action of LMTSA codes, the flow may also be in the other direction. We propose that the presynaptic neuron may generate a number of LMTSAs, possibly including microRNAs, which may be transported by the exosomal system into the synaptic cleft, and thence may be taken up by endocytotic mechanisms into the postsynaptic neuron. In that, they may exert the epigenetic actions associated with a number of different types of information processing. In the normal course of events these factors would refine the mode of action of existing circuits.

## Review of the evidence for the transsynaptic transport of LMSAs in neurons

The evidence that exosomes transport lipids, proteins, a variety of RNAs, and even DNA between cells already exists (Koles and Budnik, 2012; O'Loughlin et al., 2012; Tetta et al., 2012). Direct evidence for the existence of LMSTAs specifically in neurons is presented in the following reports. Menna et al. (2003) have shown in neonatal rats that brain-derived neurotrophic factor (BDNF) is produced by retinal ganglion cells (RGCs) and travels in an anterograde direction along the optic nerve. This results in modulation of the microanatomy of their synapses in the lateral geniculate nucleus. In studies of the developing chick brain, Von Bartheld et al. (1996) report that, neurotrophins are transported in the anterograde direction, from cell bodies to the axon terminals, and that the intact neurotrophin is released after anterograde transport, taken up and utilized by the postsynaptic neuron via axo-dendritic contacts. They conclude “These results suggest that anterogradely transported neurotrophins may play a role in synaptic plasticity and may have effects at more than one synapse beyond the initial release site.” These two reports mention anterograde transport of neurotrophins leading to transsynaptic communication, but do not specifically involve exosomes. However, exosomes would seem to be the most likely candidates to transport large molecules across the synaptic cleft. The third report provides evidence that this process actually takes place. At the Drosophila neuromuscular junction the signaling morphogenic protein Wg is transferred inside exosomes (budded from multivesicular bodies) through binding to the exosomal protein Evi. The exosome, with its Wg load, is then released from the presynaptic terminal to be taken up by the postsynaptic target, in this case muscle cells (Koles and Budnik, 2012: see their Figure 1).

The authors suggest that the site of release is probably not the active site of the synapse, which is specialized for neurotransmitter release, but in the periactive region around. There is also the possibility that transfer could be mediated by extrasynaptic mechanisms that are widely spread throughout the brain (Smythies, 2002). There is another consideration. This release at the neuromuscular junction is into the subsynaptic reticulum, which is a series of cisternae peculiar to the neuromuscular junction. However, the active site at the interneuronal synapse, which lacks any subsynaptic reticulum, is only 20 nm wide, whereas the diameter of an exosome is 50–90 nm. So there is no room at the actual synapse for any such transfer. Therefore, if such transfer takes place at the interneuronal synapse, it must do so at extrasynaptic regions. The problem is that there is no real extracellular space in the brain. The space between neurons is effectively filled by glia. Moreover, as far as we are aware there have been no electron microscope detections of structures resembling exosomes in between neurons. This suggests that the most likely pathway for the exosomes trafficking between the presynaptic neuron and the postsynaptic neuron might be via glia. There are several reports of exosomes reacting with glia (Kramer-Albers et al., 2007; Guescini et al., 2010; Frühbels et al., 2012). Oligodendrocytes activated by glutamate secrete exosomes that contain proteins, mRNAs and microRNAs. These exosomes are taken up by adjacent neurons by a clathrin-dependent mechanism (Frölich et al., 2013). Astrocytes wrap round synapses and interact with them to form what have been called “tripartite synapses” (Haydon, 2001; Gordleeval et al., 2012). To this has been added possible involvement of the intercellular matrix to form tetrapartite synapses (Dityatev and Rusakov, 2011). These offer avenues for the interneuronal transport of LMTAs and microRNAs.

Interneuronal transport of exosomes could also be conducted via gap junctions. There are extensive similarities between neurons and elongated fiber cells that make up in the interior of the ocular lens (Frederikse et al., 2012). Electron micrographs show similarities between the organization of their intracellular vesicle transport machinery and between lens fiber cell lateral protrusions and dendritic spines. Gruijters (2003) reviews evidence that intercellular vesicle transport in the lens, possibly carrying large molecules, is mediated via gap junctions. There is also the possibility that membrane-bound proteins on the exosome could bind to complementary receptors on the postsynaptic neuron that would transfer the signal to the interior by a conformational change.

## Membrane utilization dynamics

Our hypothesis places particular significance on the interneuronal transport of exosomes, constructed of lipoprotein cell membranes, in either an anterograde, or retrograde direction, or both, carried by exosomes, which consist of membrane. This process is subject to membrane utilization dynamics. In the postsynaptic neuron the process of endocytosis of receptors is a balanced dynamic process (Smythies, 2002). Upon binding most neurotransmitter, or neuromodulator molecules, the receptor is packaged into a fold of the surrounding membrane. This pinches off to form a sack that is then trafficked to the endosome system inside the cell. Here the load molecule is extruded into the endosome cavity and the sack fuses with the endosome membrane. The load molecules are then subject to a triage process. In this, damaged (oxidized) molecules are routed to lysosomes and are broken down, and their components recycled. The rest are enclosed in endosome membrane to form more sacks, which are recycled to the surface. Here the load molecule is inserted into the plasma membrane, and the sack fuses with the plasma membrane itself. In this way membranes are continually recycled so cutting the expensive process of synthesizing new membrane to a minimum. In contrast, at the presynaptic axon terminal, the process of neurotransmitter or neuromodulator release is not affected by the extrusion of membranous vesicles. The vesicle extrudes its payload at the cell surface and the vesicle is internally recycled. Again synthesis of new membrane is kept to a minimum.

Bearing these considerations in mind, if we consider the transfer of exosomes across the synaptic cleft, possibly via glia, it becomes obvious that portage in only one direction, whether it is anterograde (pre to post) or retrograde (post to pre) will lead to an unbalanced system. The donor cell will be continuously depleted of membrane, whereas the recipient cell will be continuously swamped. Furthermore, each vesicle coat is only used once, which is an enormously wasteful process. A balanced system requires approximately equal traffic in each direction. So what loads could these two sets of vesicles carry between neurons? In the anterograde direction it would be logical to suggest the load includes epigenetic factors we have suggested for the modulation of local and cell wide information processing mechanisms. But, what function would the retrogradely delivered molecules perform? What information does the presynaptic neuron need from the postsynaptic neuron? Clues are suggested by the finding by Fauré et al. (2006) that neurons grown in tissue culture (that presumably act mainly as postsynaptic neurons being composed mainly of soma) export L1 cell adhesion molecules, the GPI-anchored prion protein, and the GluR2/3 but not the NR1 subunits of glutamate receptors. The first two can act as adhesions molecules (among many other functions), which might seem appropriate, but why subunits of AMPA but not NMDA receptors? Activated AMPA receptors are endocytosed into the postsynaptic neuron but NMDA receptors are not (Lissin et al., 1999). But it is difficult to see why that should be relevant. One explanation is that this pathway simply gets rid of unwanted molecules. Another explanation might be that exosomes represent a supplementary retrograde supply route of key synaptic components to the site of activity. The anterograde route from the cell soma to its own active synapses via its own axons is often long and slow. The transport route for the same molecules from the soma of the postsynaptic neuron to the same location via exosomes would be much shorter and faster. This might explain why AMPA, but not NMDA, subunits are trafficked by exosomes. Since the NMDA receptors are not endocytosed upon activation, their life span in the membrane is much longer that the life span of AMPA receptors. Smalheiser (2007, 2009) suggests a plausible function for retrograde exosomes in terms of modulation of synaptic plasticity. This requires the retrograde exosome to carry molecules like CAM kinase II alpha and mRNAs. However, such molecules were not detected by Fauré et al. (2006). This suggests further experiments to look for them.

## Developmental factors in the relationship between epigenetic codes and neural structure

Of course, the microstructure of the brain cannot be understood solely in terms of the postnatal activity of LMTSAs of various kinds. During embryogenesis the differential modality of the cortex is determined by classical morphogens such as FGF8. Thus each area of the cortex is differentiated in the manner described by Rash and Grove (2006). These areas secrete attractant molecules that attract the appropriate axons from the thalamus. It seems therefore that the early specification of the modality of cortical areas defines the type of sensory afferents (and, therefore, sensory modality) projecting to them and not the contrary. However, this does not rule out the possibility that, later in development, sensory modality can be modulated by a change in the sensory afferent input. In this paper, we are dealing only with postembryonic stages of cortical plasticity.

The parcellation of the cortex into functionally and structurally discrete areas involves what Sur and Rubenstein (2005) describe as “… an interwoven cascade of developmental events including both intrinsic and extrinsic elements.” The cortical progenitor zone contains the information that genetically generates cortical area, whereas, later on, thalamic afferent axons, through activity dependent mechanisms, may impose further cortical differentiation via, in part, epigenetic mechanisms. Therefore, although the plastic processes occurring during critical developmental periods may be different from those occurring after deafferentation, further investigations should enable us to distinguish between them. Sur and Rubenstein describe many transcription factors, adhesion molecules, axon guidance molecules, etc., involved in cortical plasticity. They add, “The patterning centers operate in part through generating graded expression of the transcription factors that control histogenetic programs for proliferation, neurogenesis, migration, connectivity, and cell death/survival.” They conclude, “Activity operates through modulating the expression and function of almost the entire range of molecules responsible for neuronal and synaptic function.” Further reviews of this topic have been published by Krubitzer (2007) and Rakic (2009). Beaud et al. (2012) have described how rewiring in the spinal cord can explain plasticity after peripheral injuries in adult monkeys. Spontaneous electrical activity, present at the earliest stages of cortical development, can also modulate the developing cortical structure (Sur and Rubenstein, 2005).

The exosome-based system we are suggesting would have its dynamic aspects. Activity in the system would normally be sufficient to meet the needs of replacing oxidized proteins removed by the endosome triage system. Then it should become more active during learning processes to oversee the synthesis of modality-specific new proteins involved in the learning process. This reactivation may be reflected in the reported finding that rates of exosome release are increased by depolarization of the membrane. A higher rate of activation could take place after major shocks such as deafferentation and reafferentation.

## The molecular basis of binding

We define the binding problem here as defining the mechanism by which the brain combines information generated by single modality systems into multimodal operations that must underlie the unitary percepts we experience. We do not intend to cover every aspect of the physiological basis of binding, but to focus upon a new and interesting development. We admit that this section is highly speculative, because different mechanisms may contribute to modality codes in unimodal and multimodal areas. However, there is no *a priori* reason why they should not be, if not the same, at least similar. It might seem simpler to suggest that evolution would have developed two similar mechanisms to perform two similar processes in related neurons.

If unimodal afferent axons in the sensory system have such a marked effect on the functional neuroanatomy of the postsynaptic neuron, the question naturally arises—what happens when axons which belong to two (or more) different modal systems, synapse on one multimodal neuron within higher sensory cortex? Does this neuron possess [a] one “intermediate” computational system, [b] two (or more) quasi-independent systems that somehow interact or, [c] an entirely different system? We now know that nearly all of sensory cortex is multimodal in character. In “primary” sensory cortex one mode is dominant and the others operate at subliminal levels. In higher polysensory cortex the neurons integrate all the various inputs more or less equally. These questions are relevant to the question of the neural basis of “binding.”

In multisensory cortex the incoming axons may carry different modality codes and may transport the different LMTSAs associated with these codes. A modality code is defined simply by its precise content of LMTSAs. Different individual postsynaptic neurons would receive, via the exosome system, different proportions of these signaling agents. MicroRNAs can modulate a large number of functions throughout the neuron by their action on mRNAs. All this activity translates into the dynamic feature of neurons whereby many parts of the cell is being constantly replaced (Smythies, 2002). This entails that there will be a wide variety in the functional anatomy of the information processing mechanisms inside the neurons that receive these variegated inputs. For example, one neuron may receive 30 visual, 40 somatosensory, and 10 auditory inputs axons. In another these numbers may be 60, 5, and 40—and so on. Each involves a different mix of signaling proteins, mRNAs and microRNAs. In addition, each neuron will have a unique history of its activities and impacts of a wide range of neuromodulators, in addition to the LMTSAs received. Currently, little attention has been paid to the idea that each and every neuron in the brain may be unique in this way. This opens up a wide range of possible computational mechanisms (Molfese, 2011).

In most theories of the neural basis of “binding,” attention is focused on activity in the activity of groups of neurons belonging to different modalities arranged in nerve nets. It seems probable that such activity is indeed involved. However, our hypothesis adds another parameter. In a multisensory neuron significant “binding” information may also be contained with each and every individual neuron by virtue of their specific unique electrochemical make up, as we have described. That is to say, for example, that the auditory system may have one specific pattern “A” of computational functional machinery organized in part by its own particular collection of received LMTSAs. The visual system likewise may have its own specific system “B” organized in part by its own, and different, collection of received LMTSAs. In which case, a higher bimodal neuron, to which both these two neurons project, will have its own pattern “C” that organized by its own specific collection of LMTSAs. In this case we can suggest, very roughly, that “C” equals ½“A” + ½“B.”

It might be possible to estimate the degree to which the LMTSA/exosome system that we have suggested is dynamic. In other words, what is the time frame of its operation? The exosome system seems to operate in a manner similar to the synaptic vesicle system in delivering its payload into the synaptic cleft. The uptake system into the postsynaptic neuron is also likely to be quick process. The target of a given microRNA is its own specific mRNA, which likely resides within the postsynaptic area, implying that the entire operation would be quite rapid.

Another question is how the system in a multimodal neuron might react to quantitative changes in its inputs. For example, if the bimodal input to such a neuron is 50% “A” and 50% “B,” how would it react to a change in the sensory input that raises the level of one of these inputs relative to the other—say to 70% “A” and 30% “B?” This would alter the details of the modal code “AB” sent by this neuron to higher centers. Would this change carry usable information?

We can also ask whether the spike codes and LMTSA codes are translatable into each other. Since the LMTSA code is modulated by a variety of neural and synaptic events (Fauré et al., 2006; Barmack et al., 2010; Impey et al., 2010; Cohen et al., 2011; Hsu et al., 2012; Saba et al., 2012; as detailed above) it is possible that the temporal pattern of incoming spikes at a synapse that carry the afferent spike code could modulate the LMTSA/exosome system. Likewise the emerging pattern of LMTSA-induced modulation at the postsynaptic site could modulate the membrane events that lead to the spike formation and timing that generate the efferent spike code.

## Experiments to test our hypothesis

The most pressing need is to repeat the experiments, reported by Koles and Budnik (2012) in the neuromuscular junction, at synapses made by axons on postsynaptic neurons. This would answer the question whether exosomes do at the interneuronal synapse what they have been shown to do at the neuromuscular junction. This should be followed by experiments to test if and how exosomes cross such synapses, particularly to determine if astrocytes are involved. Then, detailed experiments are needed to trace the passage of the LMTS agents in the postsynaptic neuron. Experiments are indicated that would look for specific signaling proteins, mRNA, and microRNAs in axons of different modalities. It would be informative to determine the mode of action in more detail of the LMTSAs listed above that have been found to modulate neuronal function. Further experiments are needed to determine the exact mechanisms of transport and transfer of this material down axons.

## Conclusion

The impact of the idea coined by Smalheiser (2007, 2009) and Fauré et al. (2006), that LMTSAs (including microRNAs) can act as signals between neurons and play a role in information processing on cognitive neuroscience, has been limited. In fact, only one group (Koles and Budnik, 2012) has taken up this topic since. We hope that our paper will help extend further research into this important subject. Most reviews aim to present advances in scientific knowledge in their subject. In contrast, this review discloses a lamentable state of ignorance concerning a key function of the brain—the possible existence of a hitherto unrecognized widespread signaling system. It now seems probable that the afferent axons carry, not only minute-to-minute operative information in the form of spike trains, but also instructions in the form of specific large signaling molecules. It is possible that, under normal circumstances, these instructions refine the operations of the sensory, and probably other, cortex that processes this information down-stream. We have hardly any idea how this is done: nor how far down the chain of information processing this process extends. However, there seem to be some promising candidates in the field. These include axonal temporal spike trains and synchronized oscillations, and specific patterns of LMTSAs transported between neurons in the cortical hierarchy by the exosome system.

### Conflict of interest statement

The authors declare that the research was conducted in the absence of any commercial or financial relationships that could be construed as a potential conflict of interest.
